# Profile, perceptions and future expectations of medical laboratory scientists in Namibia

**DOI:** 10.4102/ajlm.v4i1.246

**Published:** 2015-08-20

**Authors:** Bruce H. Noden, Vincent Nowaseb, Cornelia de Waal-Miller, Berta E. van der Colf

**Affiliations:** 1 Department of Health Sciences, School of Health and Applied Science, Polytechnic of Namibia, Namibia; 2 Department of Entomology and Plant Pathology, Oklahoma State University, United States; 3 National Commission on Research, Science and Technology, Namibia

## Abstract

**Background:**

Public healthcare systems in sub-Saharan Africa are challenged by healthcare worker shortages, loss of trained staff and attrition to the private sector. Studies have historically focused on medical doctors, nurses and pharmacists, with limited focus on medical laboratory scientists.

**Objectives:**

This study addresses the professional perspectives and expectations of the first two classes of biomedical science students, who graduated from the Polytechnic of Namibia in 2012 and 2013.

**Methods:**

A questionnaire was developed to capture qualitative and quantitative data from fourth-year students completing their final semester. Data collected included: demographic information; students’ experience; professional expectations; and perceptions about the future of biomedical science education in Namibia.

**Results:**

Amongst the 42 of 45 enrolled students who completed the questionnaire, nearly two-thirds anticipated working in government hospitals (29%) or industry (35%), with fewer planning careers in private hospitals (12%) or academia (14%). Most expressed an interest in working abroad (64%) and/or in the capital (64%), with fewer interested in small urban areas (48%). Only 7% expressed interest in working in a rural area. Regarding their view of the future of biomedical science in Namibia, 38% responded that it was encouraging, whereas the rest responded that it was uncertain (52%), negative (2%) or unknown (7%).

**Conclusion:**

Members of the first graduating classes of Namibia’s nascent Biomedical Science degree programme reported a perceived lack of opportunity for professional advancement in the field if they remained in Namibia. Continued thought needs to be given to develop sustainable strategies and opportunities to retain Namibian biomedical laboratory scientists in Namibia.

## Introduction

Countries in sub-Saharan Africa are challenged by a lack of healthcare workers.^1^ On average, healthcare systems in African countries field less than 22.8 skilled healthcare workers per 10 000 population; far below the 59.4 per 10 000 population average documented in more developed countries.^2^ The already inadequate numbers of healthcare workers being trained are often further reduced by ’out‘-migration to other countries; it has been reported that 8000 nurses leave sub-Saharan Africa every year.^3^ This migration, commonly referred to as the “brain drain”, presents a difficult challenge for developing countries already facing substantial barriers to provide a minimal standard of healthcare services.^4,5,6^ For many healthcare workers, migration to affluent countries is driven by inadequate salaries, lack of training opportunities, limited chances for promotion, safety concerns and poor living conditions at home.^4,5,7^ It is also driven by healthcare worker shortages in developed countries.^1^

Most evaluations of the “brain drain” phenomenon have focused on medical doctors,^8,9,10^ nurses^5^ and pharmacists.^11^ Despite the important role of medical laboratory scientists in the medical diagnostic field, there is almost nothing in the current literature about the impact of this cadre’s out-migration on the health systems in developing countries.^12^ Medical laboratory scientists perform the tests, generate the preliminary reports and, together with other qualified laboratory professionals, monitor the quality of clinical laboratories. Altogether, this teamwork culminates in generating reliable results so that evidence-based diagnoses and effective patient care are delivered. The persistent shortage of medical professionals, including medical laboratory scientists, is well reported in countries such as the United Kingdom and United States.^13,14,15^ There is, therefore, an increasing possibility that the “brain drain” phenomenon documented amongst doctors and nurses could also be extended to young African laboratory technicians and technologists and medical laboratory scientists. The resulting loss of these professionals would not only undercut the quality of healthcare delivery in Africa, but will also drain the few resources available to the nascent laboratory science training programmes on the continent and may, eventually, cause them to be eliminated.^16^

Namibia has historically relied on South African universities to train its medical personnel. The country also has a long history of reliance on foreign healthcare workers in all major health-related cadres (doctors, nurses, pharmacists and medical laboratory scientists). Starting in 2008, the University of Namibia (UNAM) has offered degree programmes in Medicine and Pharmacy. In the same year, the Polytechnic of Namibia (PoN) introduced degree programmes in Biomedical Science and Environmental Health. The Biomedical Science programme at the PoN was hosted by the School of Health and Applied Sciences. Prior to 2008, the technical staff of public and private laboratories comprised either foreigners or Namibians who left the country to complete a three-year diploma in South African institutions. In 2005, the PoN was approached by local stakeholders, as well as the Ministry of Health and Social Services, to create a training programme in-country. An advisory committee developed a curriculum for a four-year professional degree programme, which would allow graduates to move into a master’s programme and, ultimately, a doctorate in laboratory sciences. The four-year professional bachelor’s degree incorporated clinical chemistry, haematology, microbiology and molecular diagnostics with other important subjects, as well as a research project. After four years at the PoN, the students complete a one-year internship in industry prior to registering as Medical Laboratory Scientists. This programme, which partnered with Cape Peninsula University of Technology (CPUT) in Cape Town, South Africa, was the first such programme in southern Africa. The curriculum and course were approved in 2007 and the first intake of students occurred in January 2008. As the programme developed, external assistance was provided by a President’s Emergency Plan for AIDS Relief (PEPFAR) development grant through the local Centers for Disease Control and Prevention office and a twinning arrangement with University of Arkansas for Medical Sciences.

The new programme began with three faculty members teaching the first cohort of students. The programme grew each year, adding new faculty members, most recruited from industry. Through time, the training laboratories were equipped for practical modules. The Ministry of Education offered study loans to students and the Ministry of Health and Social Services offered several bursaries. Whilst starting off with excitement, there were considerable challenges in the first years of the programme. Finding teaching venues in a dynamically-changing educational institution, in addition to challenges in finding faculty members, meant that practicals were not presented concurrently with theory sessions and international instructors were brought in to provide intense short practical modules. Whilst all of these have now been addressed since the programme moved into its own building in 2014, the first two cohorts of students worked through the challenges of the early years. The PoN graduated its first class of medical laboratory scientists in 2012.

Medical education is an important national investment, but the returns obtained are not always what are hoped for, or even expected.^5,7^ In Namibia, where a trained, dedicated cadre of non-expatriate healthcare workers is desperately needed, it is important for nascent healthcare professional training degree programmes to understand what students expect from the education programme in which they have enrolled; and, more importantly, what they expect from their home county’s healthcare system in terms of career opportunities, remuneration and professional advancement after graduation. Studies focused on the students, particularly in the early years of these valuable training programmes, provide Namibian educators and policy makers with valuable information about how national investments in professional healthcare education may (or may not) benefit the national healthcare system.^5,8,9,10,11^ As an initial component of this research, the objective of this study was to describe and analyse the profile of the first two classes, which graduated in 2012 and 2013, in regard to personal and educational backgrounds, experiences whilst at the PoN and future career expectations.

## Research method and design

### Materials and setting

Consecutive fourth-year, final-semester students (study size: *N* = 22, 2011; *N* = 23, 2012) were enlisted from the first two classes graduating from the PoN Biomedical Science programme in 2012 and 2013. Only fourth-year students in the programme attending a tutorial during the preparation of their Honours thesis research projects were provided an opportunity to complete the questionnaire. Those who did not attend the tutorial (*n* = 3) were not given another opportunity.

### Design and procedure

A questionnaire was adapted from Modipa and Kambisya^11^ and Nguyen et al.^5^ Prior to filling out the questionnaire, the research team went through the questions with the students to ensure understanding. All students were provided as much time as necessary for full analysis of each question and a moderator was on hand to answer any questions. The questionnaire contained closed- and open-ended questions concerning: the demographics and backgrounds of the students; their experiences and perceptions during the four years of training; and their expectations and final perceptions of the programme.

### Analyses

All data were entered into a Microsoft Excel™ spreadsheet and analysed using IBM SPSS Statistics for Windows, version 21.0 (IBM Corp., Armonk, NY 2012). For categorical data, Pearson’s χ^2^ tests or Fisher’s exact tests were used. Binary logistic regression analysis was used to assess associations between population characteristics and desire to work abroad. *P*-values of less than 0.05 were considered to be statistically significant.

#### Ethical considerations

The study and questionnaire were approved by the Institutional Research and Publications Committee of the PoN, the ethical committee of the University, as per policy [IRPC-POLY/2011/7377/539]. The respondents voluntarily completed the questionnaire and all answers were recorded anonymously. Completed questionnaires were securely organised and stored.

## Results

### Study population profile

Of the 45 students enrolled in the programme, all 42 students who were present in the tutorial completed the questionnaire. Three international students (Angola, *n* = 1; Botswana, *n *= 2) were not present the day the questionnaire was given, because they were still completing their in-service training commitments. Amongst those who responded, the majority were either women (*n* = 32; 76%) or were between the ages of 21 and 25 years (*n* = 41; 98%) (Table 1). Two respondents (5%) were married. Almost half of the Namibian students were from northern regions (*n* = 20; 47%) and a third (*n* = 14; 33%) from central regions. The majority of the students had lived in an urban area before the age of 17 (*n* = 30; 71%), but most had rural experience (*n* = 27; 64%). In addition, almost two-thirds had attended government schools in urban areas (*n* = 27; 64%) and six (14%) had private education. The proportions of students with fathers (*n* = 14; 40%) or mothers (*n* = 12; 31%) with a tertiary educational experience were similar.

**TABLE 1:** Characteristics of fourth-year students in the Biomedical Science programme at the Polytechnic of Namibia in 2011 and 2012.

Variable	Result	Respondents	
		Number	Percentage
Year graduated	2012	21	50
	2013	21	50
Gender	Female	32	76
	Male	10	24
Age	21–25	41	98
	26–30	1	2
Marital status	Married	2	5
	Not married	40	95
Number of children	None	42	100
Nationality	Namibia	39	94
	A ngola	1	2
	Kenya	1	2
	Zambia	1	2
Regions of residence†	North	20	47
	Central	14	33
	East	2	5
	West	2	5
	South	4	10
Type of area of residence before age 17	Rural	12	29
	Urban	30	71
Ever lived in a rural area?	No	15	36
	Yes	27	64
Secondary school attended	Government – urban	27	64
	Government – rural	9	22
	Private	6	14
Father – tertiary education?	Yes	14	40
	No	21	60
Mother – tertiary education?	Yes	12	31
	No	26	69

†, Includes all students who participated in the survey. North (Kavango, Ohangwena, Omusati, Oshana, Oshikoto); Central (Khomas, Otjizondjupa); East (Omaheke), West (Erongo), South (Hardap).

### Perceptions and experiences of the study population

Slightly more than half of the students learned of the programme through the media (newspaper or internet) or personal contacts (Table 2). The choice to apply for the programme was predominantly self- (*n* = 19; 42%) or family-influenced (*n* = 18; 40%). Just over half (*n* = 24; 59%) chose biomedical science as their first choice. Other students were interested in other medically-related programmes (24%), including medicine, forensics, pharmacy, dentistry and psychology, which were not yet offered in the country when the programme was initiated, or engineering (12%) and environmental health (5%), both of which were already being offered at the PoN (data not included in Table 2).

**TABLE 2:** Perceptions and experiences of fourth-year students in the Biomedical Science programme at the Polytechnic of Namibia in 2011 and 2012.

Variable	Result	Respondents	
		Number	Percentage
How learned about Biomedical Science programme	Media (newspaper, internet)	22	55
	Personal communication with others†	18	45
What choice was biomedical science?	1st	24	59
	2nd	15	37
	3rd	2	5
Why did you choose programme?	Medically oriented	31	78
	Sounded interesting	6	15
	Help others	3	8
Who helped you choose the programme?	Self	19	42
	Family	18	40
	Others	7	16
	Internet	1	2
Where did you live during your studies?	University residence	7	15
	Parents’ / relatives’ home	22	49
	Renting off-campus	18	38
	Friend	1	2
Who or what financed your education?	Bursary	15	29
	Student loan	21	40
	Parents	13	25
	University agreement	1	2
	Self	1	2
	Trust fund	1	2
What has been your main difficulty?	Finances	21	33
	Transport	12	19
	Supplies (including computer)	10	16
	Housing issues	11	17
	Psychological stress	1	2

†, Friends, family, teachers, Polytechnic of Namibia visit.

When asked why they chose biomedical science, the majority (*n* = 31; 78%) responded that it was because it was medically-oriented:

‘Always wanted to do something medically related and stay in Namibia and didn’t want Pre-Med at UNAM.’ (female, 22)

‘Interested in medical field but didn’t want contact with ­patients.’ (female, 22)

Another six (15%) chose the programme because it sounded interesting:

‘I got amazed by the name. I never knew what it really was. Went to try my luck at the pre-selection and just got in.’ ­(female, 23)

‘[*G*]ood in biology so pursued related courses. Being a scientist sounded good.’ (male, 21)

Three of the students (8%) chose the programme because they wanted to help others:

‘[*H*]elp patients in order to save lives.’ (female, 25)

‘Thought I would be more helpful and productive to work in a background career instead of an upfront one.’ (female, 22)

With regard to living situations and financing of education, most students (*n* = 22; 49%) lived with family during their four years of study, whilst others rented accommodations off-campus (*n* = 18; 38%) or used the university housing (*n *= 7; 15%). Forty per cent (*n* = 21) financed their education through student loans, whilst others were covered through bursaries (*n* = 15; 29%) or family support (*n* = 13; 25%). The studies of one student were financed through an agreement between UNAM and the PoN, whilst another was financed through a trust fund. The main difficulties experienced were financial, with related mentions of transport (taking taxis to and from place of residence), supplies (textbooks, computers) and housing.

## Future expectations and perceptions

Most students indicated that they anticipated working in a government hospital setting (*n* = 15; 29%) or industry (*n* = 18; 35%) after completion of their training (Table 3). A smaller group anticipated working in private hospitals *n* = 6; 12%) or in academia (*n* = 7; 14%). One student was focused on working in a forensic laboratory. When asked why they chose their particular settings for anticipated employment, responses could be summarised into four main areas. The first area centred on exposure to new things or new diseases, as can be seen from the following responses:

**TABLE 3:** Future expectations and perceptions of fourth-year students in the Biomedical Science programme at the Polytechnic of Namibia in 2011 and 2012.

Variable	Result	Respondents	
		Number	Percentage
In which sector would you like to work after completion of your degree?	Government hospital	15	29
	Private hospital	6	12
	Industry	18	35
	Academia	7	14
	Research lab	3	6
	Forensic lab	1	2
	Private lab	1	2
Where would you like to work after completion of your degree?	Windhoek	27	64
	Urban area, not big city	20	48
	Rural area	3	7
	Abroad	27	64
If abroad, where? (of total responses *n* = 27)	Another African country	8	30
	Europe	13	48
	North America	13	48
	Australia/New Zealand	2	7
What is the future of biomedical science in Namibia?	Bright, encouraging	16	38
	Uncertain	22	52
	Negative	1	2
	Unknown	3	7
Would you have chosen to study biomedical science again?	Yes†	10	24
	No‡	32	76
Would you encourage someone else to study biomedical science at the Polytechnic of Namibia?	Yes	21	50
	No	18	43
	Not sure	3	7
What are your future plans as a medical laboratory scientist?	Work in a laboratory	7	14
	Gain further education	29	58
	Do something completely different	14	28

†, Expected more but I like this field; ‡, Expected more, want to do something else, would have chosen something else.

‘[*E*]xposure to all types of diseases as government [*clinics*] deal with patients with all backgrounds.’ (male, 24)

‘[*E*]xposure to wider range of more severe illnesses more common in low-income populations.’ (female, 23)

The second area was focused on financial reasons or bursary repayment. The third area centred on the desire to improve one’s qualifications or personal growth and development, as shown by the following responses:

‘[*I*] believe these are the areas where my skills and qualifications will be utilised best and will also allow me to grow in my profession.’ (female, 22)

‘I have already worked in several med labs so for me to go into a different environment would be interesting and build my knowledge.’ (female, 23)

Finally, the fourth area was focused on personal professional choice (*n* = 2), such as a desire to improve the system:

‘So I can provide the best service possible which is not always done at Government hospitals.’ (female, 22)

‘[*T*]o improve industry.’ (male, 22);

personal choices (*n* = 3):

‘I don’t like hospitals and academia is not my field either.’ (female, 22)

‘I love to test water, to make sure it is sterile as this is the ­biggest need in all living beings.’ (male, 22)

‘I want to be a lecturer or training officer at PoN one day so that I can train students very well and hopefully to [*sic*] make Biomed the best course at PoN.’ (female, 23);

or altruism (*n* = 1):

‘[*I*] would like to help underprivileged people in society.’ (male, 25)

With respect to location of practice, 55% of respondents provided more than one answer. Sixty-four per cent (*n* = 27) wanted to work in the capital (Windhoek), 48% (*n* = 20) in a small urban area 64% (*n* = 27); wanted to work abroad and 7% (*n* = 3) wanted to work in a rural area (Table 3). Of those considering working abroad, 48% (*n* = 13) preferred Europe, 48% (*n* = 13) North America, 30% (*n* = 8) were considering working in another African country and 7% (*n* = 2) wanted to work in Australia or New Zealand. Reasons given for wanting to work abroad included: (1) experiencing new ways of doing things; (2) exposure to new techniques; and (3) attraction to research possibilities not currently available in Namibia.

Equal proportions of male (60%) and female (66%) students desired to work abroad. Although not statistically significant, fewer students who started the programme in 2008 (52%) desired to work abroad compared with 2009 (76%). There were no regional differences amongst those desiring to work abroad. Once more, though not statistically significant, more students with fathers (70%) or mothers (69%) with secondary- or tertiary-level education desired to work abroad compared with fathers (44%; *p* < 0.242) or mothers (43%; *p* < 0.225) with primary or unknown education (data not included in Table 3).

When asked whether they would have chosen to study biomedical science again, 24% (*n* = 10) answered in the affirmative; only half said they would recommend the programme to someone else (Table 3). Concerning their perception of the future of biomedical science in Namibia, 38% (*n* = 16) responded that that it was bright and encouraging, whereas the rest were uncertain (*n* = 22; 52%), negative (*n* = 1; 2%) or unknown (*n* = 3; 7%). Reasons given for being positive included:

‘[*B*]ecause new learning methods and training techniques are continuously developing.’ (male, 27)

‘We are needed, NIP [*Namibia Institute of Pathology*] is accredited. Things [*are*] looking better. Quality management is improving in labs.’ (female, 22)

‘[*W*]e are pioneers, so we can make changes as they need to be made.’ (female, 22)

‘I have faith in [*the*] country’s potential to develop.’ (female, 23)

‘[*M*]ore students in this field will make a difference.’ ­(female, 23)

‘[*M*]ore technologists = more development.’ (female, 24)

Most of those who responded with uncertainty focused on the lack of development in the industry and lack of opportunities arising from saturation of the market. Of the 22 uncertain respondents, nine mentioned the limited job market and three mentioned a lack of alternatives for personal growth in the industry:

‘No means of profession ladder, small market, foreigners saturate the market, not many alternatives.’ (female, 23)

‘Slow pace in Namibia and there is very little interest by current professionals to develop the profession.’ (female, 22)

‘[*The*] current situation in the lab does not provide a very bright future for a young developing biomed scientists, but change is possible.’ (female, 22)

One even mentioned a fear that positions would be replaced by automation in the near future. Those who were ’unknown‘ in their responses cited lack of experience:

‘I can’t predict the future.’ (female, 23)

‘[*N*]o comment, as [*I*] am not in the industry yet.’ (female, 22)

When asked what they anticipated doing in the future, the majority (*n* = 29; 58%) wished to further their education, whilst 28% (*n* = 14) wanted to explore other career fields and 14% (*n* = 7) planned to continue working in the laboratory.

## Discussion

This study is the first of its kind to assess the characteristics, experiences and perceptions of medical laboratory science students in the Biomedical Science training programme in Namibia. The implementation of a new health-related training programme in biomedical science in 2008 was unique for Namibia as well the region. As such, a number of foreign students, albeit a small percentage, as well as students from all over Namibia, were attracted to the programme. This novelty provided a diverse environment in which students from different cultures and socio-economic levels could interact professionally and support one another in their development. The characteristics of the first cohort of students were similar to other studies of medically-oriented students in other African countries.^8,9,10,17,18,19^ In a country where less than 1% of the adult population has a tertiary degree, one interesting component was the relatively high proportion of students whose parents had a tertiary degree (between 31% and 40%). Whilst Nguyen et al.^5^ found an association between desire to emigrate and mothers having a tertiary education, our study did not replicate this finding. This, possibly, speaks not only to their desire to work in a medically-focused profession but it could also be related to the pioneer spirit that these first two cohorts had as they worked through the challenges of a developing programme. Having parents with tertiary degrees may have provided a stabilising factor which kept them pursuing their degrees.

The results from the survey also highlighted some of the important challenges faced by students in those initial years. In fact, it was quite striking that 76% of the respondents indicated that they would not have chosen to study biomedical science again. There are a number of possible reasons for this result, some of which could be gleaned from their responses. First of all, biomedical science was not the first choice for over 40% of the respondents. Respondents seemed to be attracted to medically-oriented careers but, for almost 25%, only after they were not able to get into other health-related programmes. They may have settled, instead, for a course that they did not know much about and were now looking at a career in something they were not sure about after having invested four years. Secondly, financial difficulties, which were cited by almost one-third of the respondents, could have affected responses whilst reflecting on the questions. Whilst some explicitly cited ’financial difficulties‘, the influence of finances was observed in the other challenges mentioned, such as transport, supplies and housing. For example, transport was critical, because 38% of the respondents lived off-campus and needed to take a taxi (US $2–3/day) to get to and from campus. The cost incurred by that alone meant that some did not have funds for a midday meal but had to wait to eat until they returned home in the evening. The housing conditions in which many students lived were also difficult. Those living with relatives often lived in less-than-ideal situations, because the family members were distantly related or the students were an added burden to an already challenging family situation. This meant the students had to move often between supporting family members or put up with basic conditions that lacked running water or electricity. The challenges away from the campus often meant that classes were missed or stressful conditions were endured in order to achieve this degree. Finally, the two-year period (2012–2013) during which the respondents were finishing the programme was filled with uncertainty as the Namibian professional stakeholders, which first supported the programme, began to realise the implications of 23–24 graduates per year entering the workforce. This produced public questions concerning whether there would be enough positions for graduates. It is notable that shortly after these two groups of students graduated, the programme’s intake size was reduced from 30 to 15 to accommodate these industry concerns. Taken together, it is still intriguing that 50% of the respondents would encourage someone else to study biomedical science at the PoN. This may indicate that, whilst the programme may not have been what they had hoped for, they saw qualities that would benefit others. This aspect needs to be developed further in future studies focused on the alumni of the programme.

In addition to the challenges faced by students, this study provides insights into the motivations and future expectations of this valuable cadre of health professionals and provides data suggesting the necessity of keeping them actively engaged in their home country. Of the 42 students surveyed, 64% expressed interest in going abroad, even for a while, if given the chance. The main theme that resonated throughout their responses was that of perceived opportunity for advancement, either in experience or training. With respect to the preferred work location, respondents had a strong desire to work in urban areas or abroad. The reasons given were motivated primarily by a strong desire for opportunity and access to self-development. At that point in their training, the majority of respondents felt that the future of their profession was uncertain, mainly because they could only see job saturation in the near future as the industry is not growing fast enough to meet their needs.

This desire for self-development opportunities resounds across sub-Saharan Africa as countries grapple with the out-movement of trained medical professionals to more affluent, opportunity-providing countries. According to our results, the responses of these Namibian students to emigrate to more innovative environments in which to learn new technologies is similar to those of other African medical students,^8,9,10,17,18,19^ nurses,^5,20^ pharmacy students^11^ and medical laboratory professionals.^6,16,21^ This study provides a glimpse into yet another cadre of medical professionals in Africa who are looking for ways to better themselves and move into positions where they can earn and achieve more from their training.^5^ The fact that a majority chose the programme themselves meant they saw potential to be satisfied in this profession. To keep them in it, however, careful consideration and innovative thinking is needed, if Namibia wants to achieve its stated health development goals in the next 10 years.^20,22^

It was not surprising to find that only 7% of respondents were willing to work in rural areas. This is most likely because the majority grew up and attended schools in urban areas. Less than 30% lived in rural areas and attended rural high schools before the age of 17. Similar studies report that experience in urban areas creates a natural desire to stay in those areas.^5^ The same applies for rural experience and/or rural placement.^11,23^ Often, this motivation is fuelled by more than just the higher earning potential.^11^ In the case of these students, the majority had lived and attended school in urban areas and, as a result, desired to stay connected, bringing in the theme of opportunity and future plans once again. The fact that most covered their education via loans means they have obligations to repay those debts. Bursary arrangements often mean there is an expectation to pay back the sponsor by working for the sponsoring company for a certain number of years. Those whose parents supplemented their tuition and living expenses may also have some kind of payback expectations, which keeps the student closer to the urban context where such opportunities are available.

The equal desire of these Namibian students to work either in Europe or North America was notable. Studies have reported that students often emigrate to their colonising countries because of shared national language and education systems.^4^ Germany and South Africa both have important roles in the historical development of Namibia with both German and Afrikaans being spoken by large portions of the population. Now that Namibia has developed medical training facilities in a number of disciplines, it will be important to monitor student emigration patterns.^24^

### Limitations

One of the limitations of the study was the sample size. Students in other years could have been included in the study group but the designers of the study felt it was important to capture the responses of the first two years of pioneering students in order to identify ways to improve the program. This served as a form of assessment, not only for the programme but also for the industry as a whole, as these students represented a cohort of highly-qualified Namibians who did not leave the country to study abroad but rather persevered through challenges and difficulties. Another limitation is the use of a cross-sectional approach to capture the perceptions and intentions of these students. As a questionnaire only monitors a student’s opinion on a particular day, it can only be interpreted as an idea of how they perceive their future at that point in time.

## Conclusions

As the first study to assess the characteristics, experiences and perceptions of students in the nascent medical training programmes in Namibia, these results provide a glimpse into yet another cadre of medical professionals in Africa who are looking for ways to better themselves and move into positions where they can earn and achieve more from their training. The diversity of their backgrounds, challenges faced as they pursued training in a developing programme and concerns for the future have common threads with other health-related cadres in developing countries. The desire for meaningful engagement and opportunity and the uncertain view of how their training will integrate with the national programme means that merely having new medical training programmes in Namibia is not enough. Continued thought needs to be given by the stakeholders and government entities that originally envisioned the programmes in order to develop strategies and opportunities which will keep these professionals meaningfully engaged in the country.
